# Digital twins: a new paradigm in oncology in the era of big data

**DOI:** 10.1016/j.esmorw.2024.100056

**Published:** 2024-07-16

**Authors:** L. Mollica, C. Leli, F. Sottotetti, S. Quaglini, L.D. Locati, S. Marceglia

**Affiliations:** 1 Medical Oncology Unit, Istituti Clinici Scientifici Maugeri IRCCS, Pavia, Italy; 2 Department of Internal Medicine and Medical Therapy, University of Pavia, Pavia, Italy; 3 Department of Electrical, Computer and Biomedical Engineering, University of Pavia, Pavia, Italy; 4 Department of Engineering and Architecture, University of Trieste, Trieste, Italy

**Keywords:** digital twins, clinical oncology, big data, artificial intelligence, cancer patient

## Abstract

Recent advancements in health care digitalization opened the collection and availability of big data, whose analysis requires artificial intelligence-based technologies to facilitate the development of predictive tools supporting decision making in clinical practice. In this context, the idea of constructing ‘digital worlds’ to evaluate the performance of such novel tools becomes more attractive. Digital twins (DTs) are ‘digital objects’ characterized by a bi-directional interaction with their ‘real-world counterparts’. DTs aim to enhance predictions further by leveraging both the predictive capabilities of digital simulations and the continuous updating of real-life data—ideally incorporating clinical records, multiomics data, and patient-reported outcomes. DTs can potentially integrate these diverse data into virtual models applicable across pre-clinical to clinical studies. Running simulations *in silico* on cancer cells or cancer patients’ DTs can provide valuable insights into cancer biology, clinical practice, and health care education, with the added value of reducing costs and overcoming many common limitations of current studies (limited number of variables, challenges in recruiting patients with rare tumors, lack of real-life feedback). Despite their significant potential, DTs are still in their infancy, facing numerous unsolved technical and ethical challenges that hinder their application in clinical practice.

## Highlights

• Big data and AI-based technologies can contribute to the development of predictive tools in the oncology field.
• DTs can be used to run simulations *in silico* and are powered by a continuous update from real-life data.
• Technical and ethical challenges still hinder the application of DTs in the clinical practice.

## Introduction

In the past decades, technological advancements boosted health care digitalization, thus opening the way to the collection of ‘big data’. This holds promise for facilitating the widespread development of predictive tools to support decision making in clinical practice. As data are not sufficient to produce knowledge *per se*, their analysis requires the application of methodologies such as machine learning (ML), deep learning (DL), or broadly artificial intelligence (AI), the latter being an umbrella term including a multitude of technologies with distinct characteristics and operating models. In the past few years, AI has been mainly applied to image interpretation and clinical decision support in medical settings, including oncology.^1^

As data availability grows, the idea of building ‘digital worlds’ becomes more attractive, as shown by the increasing trend toward anticipating possible application scenarios of the metaverse in medicine: from 9 papers retrieved in PubMed using the key word ‘metaverse’ in 2021, now the same search shows almost 400 papers. ‘Metaverse’ is a fluid concept, spanning from persistent virtual reality to augmented reality, founded on the general basic idea of interaction of ‘real’ users inside digital environments in real time. In health care, the metaverse can potentially be applied to telemedicine or medical training, including surgery in virtual environments.^2^ The concept of metaverse is often associated with the concept of digital twins (DTs).

DTs are digital models of real objects that serve as virtual counterparts employed for specific tasks. DTs are characterized by connectivity and bi-directional interactions with the real object. The continuous update with real-life data is used to dynamically optimize the predictive performance of the DT. DTs have therefore the potential to support decision making by providing reliable projections, based on multimodal and multiscale ‘big data’ collected from patients.^3^

In this review, we address the development and the challenges of DT technology, exploiting its potential applications in oncology.

## The digital twin concept

The DT concept was originally developed in the industry field, referring to digital models of real-life products used to simulate interactions and test their performance in a quicker, cheaper, and more flexible way than in the real world.^4^^,^^5^ In 2010, the National Aeronautics and Space Administration (NASA) proposed a formal definition of a DT as an “integrated multi-physics, multi-scale, probabilistic simulation of a vehicle or system that uses the best available physical models, sensor updates, fleet history, etc., to mirror the life of its flying twin”.^6^

### How to construct digital twins

There are three necessary components to build a DT, independently of the field of development: a physical product, its digital representation, and the exchange of information between them.^7^ The creation of a DT is structured through three major building blocks: (i) connectivity (bi-directional connection block) between the DT and the real world; (ii) collection and management of proper input data (data collection block); (iii) construction of the digital model (modeling block). The complexity of a DT depends on its degree of fidelity to the physical object, the frequency of data updates, and the algorithms used in the digital model.^8^

**Figure 1:**
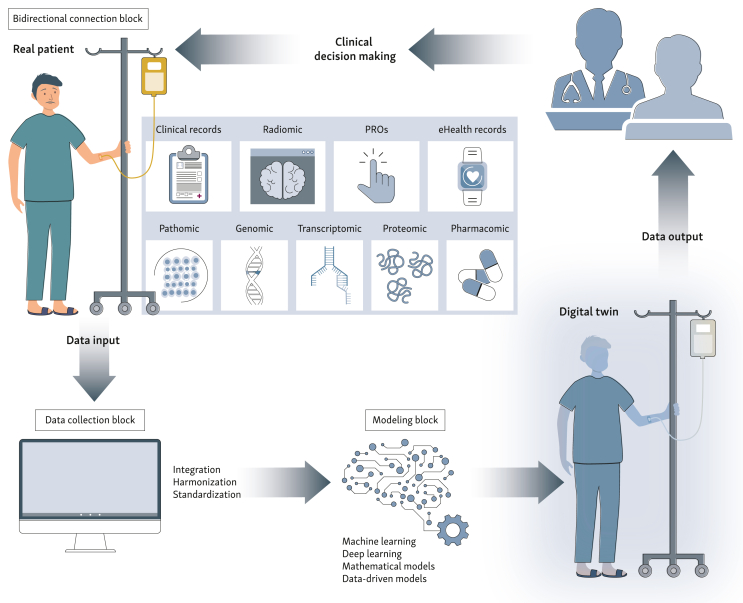
**Cancer patient DT construction process.** The cancer patient DT is built from input data derived from different sources (clinical records, PROs relating to quality of life, electronic health records obtained from wearable devices, and multi-omics data, including radiomic, pathomic, genomic, transcriptomic, proteomic, and pharmacomic). Data inputs are collected and formatted in usable data through a process of integration, harmonization, and standardization. The modeling block realizes the digital model of the physical patient. Models can be of different types (i.e. ML- or DL-based models, mathematical models, data-driven models) and produce output data (i.e. prediction of a clinical outcome) according to DT purposes. Output data can be used to inform the clinical decision making, producing a change in the real patient. This realizes a real-time, dynamic, bi-directional information exchange between the DT and the real world, allowing to continuously optimize the predictive performance of the DT. Figure created with BioRender.com. DL, deep learning; DT, digital twin; ML, machine learning; PROs, patient-reported outcomes.

As shown in Figure 1, input data come from several sources, both from the clinical domain and the patient/caregiver domestic domain. The heterogeneity of systems, poor adoption of semantic standards, and the existence of data silos act as barriers to data integration and sharing. Transforming data into usable information requires data harmonization and integration. This includes being able to manage different inputs, formatting data in such a way that they can be retrieved and used, ensuring that the different actors associate the same meaning to the same term, and ensuring data quality and control, as summarized in the FAIR acronym (Findable, Accessible, Interoperable, Reusable), which is widely used to address data quality for integration.^9^ Large initiatives and cross-national efforts, such as Observational Health Data Sciences and Informatics (ODHSI), are in place to support this transition from data to ‘usable’ data, creating the ground on which future data-driven technologies, such as DTs, can be implemented.

The modeling block realizes the digital representation of the physical object. Models can vary in type, ranging from those built through mathematical or physical/biological principles to those built through data-driven or statistical methods, as well as models based on ML, DL, or other AI techniques. The modeling block produces an output that depends on the DT purpose (e.g. prediction of a therapeutic outcome after a named intervention). The output may be visualized in a clinical dashboard or shared with the care team to inform the clinical decision-making process. This realizes the bi-directional information exchange between the DT and the real world: the decision, based on DT output, produces a change in the DT inputs that, in turn, will be processed by the modeling block with a new output.

The connectivity block is necessary to ensure that the heterogeneous input data reach the DT, while the DT outputs reach the real world. The availability of real-time synchronization techniques as well as the integration with the Internet of Things (IoT) networks are recognized as facilitators in the adoption of DT technology^10^ (The IoT refers to the interconnection of heterogeneous devices that collect and exchange data with other devices over the Internet or other communication networks. With IoT data are transferable over a chosen network without requiring human-to-human or human-to-computer interactions.^11^). However, it is important to note that connectivity represents a vulnerability point for security and cybersecurity, which should be properly addressed by DT developers.

Proper life-cycle maintenance of the DT should be planned from the beginning and should consider technology changes (e.g. connectivity updates, input data generator modifications), regulation changes [e.g. certification requirements according to reference institutions, such as regulation (EU) 2017/745 of the European Union], or model changes (due to the availability of new data or knowledge they may trigger a DT update).^12^

## Digital twins in cancer medicine and predictive oncology

In recent years, DT technology has spread in health care and biomedical research where various DT types can be used to digitally replicate cells, tissue, organs, and even patients, both healthy and diseased.^13^ In 2020 Croatti et al. outlined how DTs can be applied to health care, not only for physical computational assets, but also for (pre-)clinical experiments and care processes.^7^ In oncology, DTs are emerging as predictive tools able to boost precision oncology and facilitate personalized treatment approaches.

In Table 1 we summarize potential applications of DT that are currently under evaluation in the field of clinical oncology.

**Table 1:** Potential application of digital twin technology in oncology

**Decision-making support systems in clinical practice** Developing novel screening programs tailored to specific population risk factors Supporting physician in choosing the treatment strategy associated with the highest probability of survival Predicting treatment-related toxicity risk according to patient characteristics Monitoring the impact of the treatment on patient-reported outcomes and quality of life Early identification of resistance for prompt treatment escalation Simulation of invasive procedures to predict outcomes and safety
**Clinical trial design** Accelerating accrual and reducing cost enrolling digital patients Facilitating the enrollment of under-represented subgroups (e.g. brain metastases, comorbid patients, different ethnicities) Expanding cohort with digital patients to facilitate clinical trials on rare tumors
**Health care education in cancer care** Virtual clinical case simulations for interactive learning Facilitating remote learning for students working in small hospitals and rural areas

### Digital twins to support patient decision making

In the clinical setting, the current approach to identifying novel therapeutic strategies is based on the result of large clinical trials, which inherently have limitations in capturing the unique biological features of individual cancer patients.^14^ Clinical trials include homogeneous and ultra-selected populations and explore a limited number of treatment combinations, sequence, and schedules, which do not often mirror our real-world patients and do not address all the questions arising from daily clinical practice.^15^

In this scenario, one of the biggest challenges for DTs in oncology is incorporating the biological heterogeneity of cancer patients. DTs should integrate data from different domains collected from clinical records and wearable electronic devices: (i) patient domain (i.e. gender, ethnicity, comorbidities, polypharmacy, diet, smoking history); (ii) tumor domain (genomic, proteomic, transcriptomic, pharmacomic, pathomic, radiomic, metabolomic); (iii) environment domain (exposure to pollution, chemical agents, infective agents). All these data will constitute the framework of cancer patients’ DTs, from which mathematical models or ML/DL algorithms will draw to dynamically predict *in silico* tumor behavior and clinical response to different treatment options.^16^

A cancer patient DT framework powered by a continuous life cycle for shared decision making can ideally be able to reproduce changes across different temporal and dimensional scales, ranging from intangible transitions occurring in nanoseconds at the molecular level to macroscopic changes arising at the population level across many years.^14^ The model can be used to carry out virtual experiments in different therapeutic scenarios, thus advising decision making, while updated real-patient data should be used to guarantee continuous learning and model optimization. Continuous quality controls are needed to reduce the risk of learning from biased data.^17^

This technology would be particularly valuable for gathering data on rare cancers, where conducting large randomized clinical trials is extremely challenging. By accounting for realistic range parameter variations, DTs can be created to expand the cohort with virtual patients, potentially enabling quicker and more cost-effective attainment of sufficient statistical power.^18^ We do not know of any published work on DT applied to *in silico* clinical trials.

On the contrary, some preliminary experiences in the field of DT applied to decision-making support systems in oncology are already available in the literature. For instance, Tardini et al. proposed a clinical decision framework based on a deep Q-learning (DQL) DT to support the identification of the optimal sequential treatment in patients with oropharyngeal squamous cell carcinoma. In this project, coupled DT (treatment decision DQL-DT and the patient DT) were constructed, trained, and tested on a dataset of 536 patients with the aim of guiding a decision-making workflow tailored to a single patient. The model demonstrated high accuracy in long-term outcome and toxicity prediction, significantly improving the predicted survival and dysphagia rate in the test set.^19^

In 2023, Chaudhuri et al. developed DTs for high-grade glioma to support risk-aware decision making in selecting the optimal radiotherapy plan. The DT was first fed with clinical data extracted from literature, and then it was personalized using Bayesian model calibration for including patient-specific data [including magnetic resonance imaging (MRI) data]. This calibrated model was used to predict tumor shrinkage and radiotherapy-induced toxicity. An *in silico* cohort of 100 patients with high-grade glioma was created and tested with different strategies (standard-of-care radiation treatment and personalized approaches). In this model, DTs offered a spectrum of optimal solutions, pinpointing patients who could benefit from lower radiation doses and those with more aggressive disease requiring intensified treatment regimens.^20^

DTs can also support decision making in the simulation and optimization of invasive procedures. Ahmadian et al. proposed a DT framework for simulating both the vertebroplasty procedure and its impact on mechanical stability in patients with lytic vertebral metastases. To simulate the procedures, computational fluid dynamics was employed to predict the morphology of the vertebra before and after cement injection. A data co-registration algorithm was then employed to convert the initial model to a high-fidelity continuum damage mechanics model of the ‘digital’ vertebra. The model can be used to simulate the vertebroplasty procedure and to forecast its impact in stabilizing vertebrae carrying metastatic lytic lesions.^21^

### Image-guided digital twins in oncology

Biomedical imaging is quickly moving from being a diagnostic tool in clinical practice to being fully integrated into the complex framework of personalized precision oncology, as it can provide information on pathophysiological, metabolic, and molecular features of the tumor.^22^ Recent advancements in high-throughput extraction of quantitative features from imaging and their subsequent transformation into digital data useful for clinical decision support systems led to the birth of a new discipline, radiomics. Therefore, cancer patient DTs need to be informed also by radiomic-derived data.^23^

Firstly, imaging may provide observational data to construct and calibrate the initial geometry of DTs, linking the ‘real’ object (i.e. tumor mass of a specific patient) to its ‘digital’ counterpart, while models may predict tumor biophysical dynamics and treatment response. Longitudinal imaging measurements can be used to validate the model in a patient-specific manner, thus implementing the bi-directional dataflow and enabling the dynamic redefinition of the DT state. Imaging-guided DTs may help to identify personalized follow-up schedules according to tumor-specific biology or guide treatment (de-)intensification through early detection of tumor response or treatment resistance, respectively.^24^

However, the limited availability of comprehensive imaging assessment (due to single-center logistics, high costs, and elevated commitment for patients) represents the most meaningful barrier to the implementation of radiomic-informed DTs.

In this field, Wu et al. first proposed image-guided DTs informed by computed tomography, MRI, and positron emission tomography scans of breast tumors. These three modalities allow the integration of information on tumor morphology, tissue cell density, metabolism, vascular perfusion, and permeability. Mathematical models were then used to make patient-specific predictions of response to a range of different treatment plans (i.e. response to chemo-immunotherapy in the neoadjuvant setting).^25^ Similarly, Jackson et al. developed a patient-specific mathematical model of glioblastoma. MRI data were used to derive information about proliferation and invasion. The model was used to predict tumor growth, to forecast response to radiotherapy, and to select patients who could benefit from different surgery strategies.^26^

### Application to health care education in cancer care

DTs have the potential to boost health care education by providing realistic and interactive learning experiences. Young oncologists can use DTs to simulate different moments of cancer patient care, from diagnosis to treatment planning, and toxicity management. A large number of simulations can be run in a short time, allowing the student to learn also from rare clinical situations wherever he/she works.

DTs may contribute to continuous learning by providing updated information, new case studies, and the latest research findings. In addition, when in-person training is not allowed, DTs can facilitate remote learning. Students and health care professionals can access virtual oncology laboratories and patient cases from anywhere, recognizing a flexible and accessible education.^27^^,^^28^

## Unsolved problems and challenges

DTs are still in their infancy and several unsolved problems and challenges should be taken into consideration before they can become a reality. The challenges identified up to now are not only technical, but also ethical (Table 2).^29^

**Table 2:** Unsolved problems and challenges to digital twin technology development

**Technical challenges** Collecting high-quality and high-volume data derived from diverse populations Data integration, harmonization, and standardization Ensuring secure and adequate data storage Accessing high-performance computing resources
**Modeling challenges** Optimizing mathematical and AI-based models according to relevant outcomes Reducing risk of learning from biased data Improving the continuous update of data from real-world resources
**Ethical challenges** Safeguarding data privacy and security Ensuring equitable representation of diversity in terms of gender, ethnicity, and social background Adhering to national and international regulatory laws

AI, artificial intelligence.

Among the technical challenges, some are related to data collection, and others to modeling and connectivity. The generation and acquisition of multimodal, heterogeneous, high-volume, and high-quality data may pose challenges in terms of integration, data representation, and data storage. Quality assurance, data safety, and privacy security are challenging aspects to ensure when real-world data are used as inputs for digital models. Apart from concerns about the safety of digital health data, issues of consent related to the future use of data are currently under debate.^30^^,^^31^

Another relevant aspect regards the availability of sufficient computational power to allow the processing of a huge amount of data and complex algorithms. Improved access to high-performance computing resources should be therefore implemented to allow full exploitation. In addition, there is an environmental sustainability evaluation to be considered: data storage and processing increase the energy demand, and this drawback should be considered in the risk–benefit evaluation of DT technology.

Among the ethical challenges, it is important to mention the need to ensure that data are representative of diversity. This implies the ability to represent both healthy and diseased individuals, as well as diverse populations (e.g. low- and high-income countries having different levels of health digitalization), to avoid simulation biases.^32^

A final consideration pertains to the necessity of a skilled workforce, encompassing both users and implementers. The former requires comprehensive knowledge of DT technology to ensure proper adoption and sufficient awareness levels, thereby enhancing patient safety and model performance. The latter necessitates not only technical skills for the implementation process, but also an interdisciplinary approach to ensure the quality of the final product. To achieve this, AI lessons need to be integrated into degree programs for medicine, nursing, and other health care professions.

## Conclusions and future perspectives

DT technology presents a significant opportunity in oncology. Currently, its potential applications in cancer research range from pre-clinical research to decision making in clinical practice, to health care education.

Despite their great potential, DTs are still in their early stages, facing several barriers. Technical and ethical challenges still hinder their application in clinical practice, with ethical data collection and data harmonization being a primary obstacle. Without addressing these challenges, DT models will always lack reliability.

Interdisciplinary collaboration across various fields, such as medicine, engineering, physics, mathematics, and law, will be crucial for future DT development. We hope that our perspective will motivate further discussion and inspire more collaborations among experts from different fields.
